# Uncovering leading compounds for alzheimer’s disease treatment: mendelian randomization and virtual screening insights into plasma protein modulation

**DOI:** 10.1186/s40659-025-00598-2

**Published:** 2025-04-05

**Authors:** Xiaohan Sun, Xiaofei Hu, Jianming Wei, Haoyu An

**Affiliations:** 1 School of Science, Hong Kong University of Science and Technology, Hong Kong, People’s Republic of China; 2 Department of Nuclear Medicine, Southwest Hospital, Third Military Medical University, Chongqing, China; 3 Central Diagnostics Laboratory, University Medical Center Utrecht, University Utrecht, Utrecht, The Netherlands; 4 Population Health Sciences Institute, Newcastle University, Newcastle upon Tyne, UK

**Keywords:** Alzheimer’s disease, Plasma protein, Drug targets, Bioinformatics, Mendelian randomization analysis

## Abstract

Alzheimer's disease (AD) is a neurodegenerative disorder influenced by both genetic and environmental factors. Identifying therapeutic targets and interventions remains challenging. This study utilized Mendelian Randomization (MR) to investigate causal relationships between plasma proteins, lifestyle factors, and AD, along with virtual screening to identify potential drug compounds. A two-sample MR analysis assessed associations between plasma proteins, identified through genome-wide association studies (GWAS), and AD risk. Co-localization analysis (CA) confirmed the overlap between protein expression and AD susceptibility loci, and reverse MR ruled out reverse causality. A protein–protein interaction (PPI) network was constructed to explore therapeutic targets, followed by virtual screening to identify small-molecule inhibitors for selected proteins. The analysis found significant associations between eight plasma proteins and AD, with five proteins (GSTP1, BIN1, Siglec-3, SERPINF2, and GRN) showing strong evidence of involvement in AD pathogenesis. Virtual screening identified six compounds as potential inhibitors of GSTP1 and four compounds as potential inhibitors of BIN1. Furthermore, MR analysis of lifestyle factors, such as dietary behaviors and smoking cessation, indicated they may influence AD risk through their effects on specific proteins. These findings offer novel insights into the genetic mechanisms underlying AD and highlight the potential of combining MR with virtual screening to identify therapeutic targets. The study also suggests that lifestyle modifications could offer alternative prevention and treatment strategies for AD. Future research should focus on the experimental validation of the identified compounds and further explore the mechanisms linking lifestyle factors to AD.

**Supplementary Information:**

The online version contains supplementary material available at 10.1186/s40659-025-00598-2.

## Introduction

Alzheimer's disease (AD) is a progressive and irreversible neuro degenerative disorder that affects cognition, function, and behavior [1]. It is classically characterized by two hallmark brain pathologies: β-amyloid plaque deposition and neurofibrillary tangles of hyperphosphorylated tau [2]. AD is the leading cause of dementia, responsible for over half of all cases. Alzheimer Disease International estimated a global prevalence of 50 million cases and approximately 10 million new cases of dementia in 2015. By 2050, the number of dementia patients is projected to reach 152 million globally, with the largest increase anticipated in developing countries [3]. Heritable factors contribute to 60–80% of the risk of developing AD. PSEN1, PSEN2, APP, and SORL1 are identified as causative genes, while one or two alleles of APOE4 are recognized as risk factors [4].

The pathogenesis of AD is multifaceted. At the protein level, a network module associated with glucose metabolism has emerged as one of the most significantly linked to AD pathology and cognitive impairment. Proteins from this module are elevated in cerebrospinal fluid during the early stages of the disease [5]. Additionally, plasma circulating proteins may serve as markers of vascular dysfunction, a component long assumed essential to AD pathophysiology [6]. Recently, genome-wide association studies (GWAS) have gained significant attention. For example, a multicenter GWAS identified five loci with significant genome-wide associations with cerebrospinal fluid (CSF) profiles, two of which were novel: rs145791381 (inflammation) and GRIN2D (synaptic functioning) [7]. Protein quantitative trait loci (pQTLs) are genetic regions associated with changes in protein expression levels. Trans-pQTLs tend to be tissue-specific, while cis-pQTLs are more likely to be tissue-shared. Previous studies have examined the associations between cis-pQTLs and various conditions, including inflammatory bowel disease [8], IgA nephropathy [9], heart failure [10], ischemic stroke [11], type 1 diabetes [12], and psychiatric disorders [13]. However, the associations between cis-pQTLs and AD remain largely unexplored. Understanding these associations could help elucidate the complex mechanisms of this chronic neuro degenerative disease and aid in the development of targeted therapies.

MR could serve as a valuable method to evaluate causal relationships between cis-pQTLs and AD, addressing common limitations in classical epidemiological studies, such as confounding factors and reverse causation [14]. MR utilizes genetic variants specifically associated with a putative exposure as instrumental variables, allowing for inferences about the causal effect of that exposure on an outcome [15]. Due to the random assortment of alleles at conception, the distribution of genetic variants associated with a particular exposure is largely independent of confounding factors in conventional observational analyses [16]. Therefore, MR estimates are less influenced by environmental confounding factors and can provide more reliable insights into causal relationships between risk factors and disease outcomes compared with classical epidemiological studies. Moreover, since an individual genotype is determined at conception and remains unaffected by subsequent disease outcomes, the direction of causation is inherently from the genetic variant to the trait of interest, eliminating the potential for reverse causation [14]. Consequently, MR can be employed in this study to examine the causal effects between plasma circulating proteins (exposure) and AD (outcome).

Computer-aided virtual screening (VS) is an emerging and powerful method for identifying potential drug candidates from large chemical libraries. It utilizes computer-based algorithms to predict the binding affinity of compounds to a target protein without requiring physical experiments [17]. This approach significantly reduces the time and costs associated with traditional drug discovery processes. VS techniques can be broadly categorized into three types: ligand-based, structure-based, and pharmacophore-based screening. Ligand-based methods focus on the properties of known active compounds, while structure-based methods require the three-dimensional (3D) structure of the target protein. Pharmacophore-based screening, by contrast, identifies the essential features of a molecule necessary for the desired biological activity. The process begins with preparing a database containing thousands or even millions of chemical compounds. These compounds are then subjected to various filters and scoring functions to rank them based on their predicted interactions with the target. The top-ranked compounds are subsequently selected for evaluating their binding patterns with target proteins.

In addition to pharmaceutical therapies, lifestyle factors such as dietary interventions, alcohol consumption, and smoking may play significant roles in the prevention or treatment of AD. Nutrient-dense, healthy dietary components are recognized for their ability to modulate the immune system and potentially alter the neuroinflammatory processes linked to the progression of AD and cognitive decline [18]. These dietary modifications and nutraceuticals may be effective strategies for halting the onset of AD or preventing cognitive decline [19]. However, some well-known dietary interventions remain controversial. Long-chain omega-3 polyunsaturated fatty acids (PUFAs) have been associated with a reduced risk of cognitive impairment in individuals without dementia [20]. However, a randomized, placebo-controlled trial reported that long-term use of omega-3 PUFA supplementation, with or without multidomain intervention, had no significant impact on cognitive decline over more than three years [21]. Furthermore, the relationship between alcohol consumption and AD varies depending on the amount consumed. Low to moderate ethanol concentrations have been reported to protect against β-amyloid (Aβ) toxicity in hippocampal neurons, while excessive ethanol intake increases Aβ accumulation and Tau phosphorylation [22]. The association between smoking and AD is supported by Level B evidence, suggesting a weak correlation [23]. Investigating the relationships between lifestyle factors and AD-related proteins may provide novel insights into the regulatory mechanisms underlying lifestyle interventions.

The primary objective of this study is to identify plasma circulating proteins as potential therapeutic targets for AD and to virtually screen new small-molecule drug candidates. The secondary objective is to explore the relationships between common healthy lifestyle factors and the AD-related proteins identified in previous studies.

## Methods

### Study design

This study initially explored the association between plasma circulating proteins and AD using a two-sample MR analysis. To verify the causality of this association, we employed CA to evaluate the spatial overlap between circulating proteins and susceptibility to AD. A reverse MR analysis was conducted to exclude the possibility of reverse causality. Subsequently, we performed protein interaction network analysis to identify potential protein interactions and, combined with drug efficacy evaluation, prioritized potential therapeutic targets. Based on the structure of the selected target protein, VS was conducted within a library of small molecule compounds. Finally, using systematic MR, we analyzed the relationship between healthy lifestyle factors and AD-related proteins, aiming to identify key proteins that could serve as targets for lifestyle interventions.

### Data sources

#### Acquisition of cis-pQTL exposure data

We obtained high-quality proteomic data for 4907 proteins and their corresponding target genes from a large-scale GWAS conducted on 35,559 Icelandic individuals [24]. Measure using 4907 aptamers from SomaLogic, standardize data to correct for technical bias and batch effects. Potential regulatory variations have been identified, including cis pQTLs (located near coding genes) and trans pQTLs (located far from genes), highlighting their association with complex diseases such as cardiovascular and metabolic disorders. Genotyping was performed using Illumina chips, covering both common and rare variations, and strict quality control and imputation were performed to ensure data integrity [24]. To address the issue of linkage disequilibrium (LD), SNPs were pruned to ensure independence. Cis-pQTLs meeting the following criteria were prioritized as instrumental variables for MR analysis: 1) located within a ± 1 Mb window of the gene transcription start site; 2) genome-wide significance (P < 5 × 10^− 8); 3) linkage disequilibrium independence (r^2^ < 0.1); and 4) clumping with a 10,000 kb window. This robust approach identified many cis- pQTLs as key regulatory variations (Supplementary Table 1).

#### Acquisition of lifestyle factors exposure data

Data on lifestyle factors was obtained from the IEU open GWAS project and systematically included variables such as alcohol intake, meat intake, cooked vegetable intake, green tea intake, bread intake, crisp intake, battered fish intake, cheese intake, cereal intake, dried fruit intake, fruit smoothie intake, sweetened cereal intake, oily fish intake, milk intake, water intake, sleep duration, and smoking status (Supplemental Table 2). The GWAS data was obtained online by R software using the “TwoSampleMR” package (0.6.6) based on the ID provided in Supplemental Material 2, under the condition of P < 1 × 10^− 5, r^2^ < 0.001, clump = 10000kb.

#### Acquisition of AD outcome data

The phenotype based on health registration (endpoint) in Finnish data is created by combining data from one or more national health registrations, primarily using classification codes from the International Classification of Diseases (ICD) and Anatomical Chemotherapy (ACT). For the entire GWAS, over 2800 endpoints were initially constructed by combining data from different health registrations, including discharge registration, prescription drug purchase registration, and cancer registration. To study the genetic ancestral data of 224,737 FinnGen participants through genotyping quality control, FinnGen data was combined with 2504 Phase 3 reference samples from the 1000 Genomes Project, and principal component analysis (PCA) was used to identify FinnGen participants with non-Finnish genetic ancestors. Research has found that most participants have extensive Finnish ancestry; Out of 224,737 outliers, 3676 (1.63%) were removed. In data processing, 5780 duplicate items and monozygotic twins (one randomly removed per pair), as well as genetic population outliers, were removed, and a set of approximately unrelated individuals was established, where any relationships between pairings were at the third or higher level. A total of 156,977 independent individual data were obtained for calculating PCA, and 61,980 related individual data were projected onto these principal components (PCs). AD is defined as a progressive, neurodegenerative disease characterized by loss of function and death of nerve cells in several areas of the brain, leading to loss of cognitive function [25]. AD patients were determined based on ICD, 10th Revision (ICD-10). AD data was obtained from the latest FinnGen R11 release, which includes 11,755 European cases and 441,978 European controls [26].

#### Database for searching for protein–protein interactions

The search tool for the retrieval of interacting genes/proteins (STRING) database (version 12) was prepared to collect information on protein–protein interactions.

#### Compounds library for virtual screening

The Chemdiv 3D-Pharmacophore Based Diversity Library, containing 52,000 compounds, was set up for virtual screening.

### Statistical analysis

All MR-related statistical analyses were conducted using R software, version 4.3.2.

#### Primary MR analysis

MR is a method that uses genetic variations as instrumental variables to evaluate the causal relationship between exposure factors and outcomes. In the two-sample MR, the exposure and outcome data come from different independent samples, which avoids the influence of measurement errors in the same sample and enhances statistical efficiency. MR has three major hypotheses: correlation hypothesis, independence hypothesis, and exclusivity hypothesis. We chose SNPs (P < 5 × 10 ^ − 8) and F > 10 as instrumental variables to ensure the robustness of the instrumental variables. The instrumental variables are not related to confounding factors. To minimize horizontal pleiotropy, we only use genetic variations in the cis region. Further, evaluate the validity of this hypothesis through heterogeneity testing and bidirectional MR analysis (to detect reverse causality). To verify the effectiveness of instrumental variables, we conducted weak instrumental testing (F-statistic > 10) and evaluated potential level pleiotropy using MR Egger regression. The results showed that there was no horizontal pleiotropy in the positive results.

In this study, the 'TwoSampleMR' R package was used to perform MR analysis of cis-pQTLs and Alzheimer’s disease. For cis-pQTLs with only one SNP, Wald ratio results were used as the standard. For cis-pQTLs with more than one SNP, inverse-variance weighting (IVW) was the preferred method [27]. Odds ratios (OR) for increased AD risk were expressed per standard deviation (SD) increase in plasma protein levels. False discovery rate (FDR) correction was applied to adjust for multiple testing, with a threshold p-value of 0.05 used to prioritize results for further analysis.

#### Co-localization analysis

Bayesian CA was employed to assess the probability that two traits share the same causal variant. This method provides the posterior probability for five hypotheses regarding whether a single variant is shared between two traits. The posterior probability of hypothesis 0 (PPH0) represents that SNPs within the CA region are not related to either trait. The posterior probability of hypothesis 1 (PPH1) represents SNPs within the CA region that are related to the first trait but not to the second trait, while the Posterior probability of hypothesis 2 (PPH2) represents SNPs within the CA region that are related to the second trait but not to the first trait. The posterior probability of hypothesis 3 (PPH3) represents a relationship between SNPs and two personality traits within the CA region but not the same locus. The posterior probability of hypothesis 4 (PPH4) represents that SNPs and two personality traits within the CA region are related and share the same locus [27]. We used the 'coloc' R package to investigate whether the identified AD-related proteins and corresponding AD were associated with common causal variants and to distinguish confounding linkage disequilibrium (LD). We tested PPH3, where both the protein and AD are associated with the region by different variants, and PPH4, where both are associated with the region by shared variants. Strong evidence of CA was considered when the posterior probability of PPH4 exceeded 0.75 in different windows or when PPH3 + PPH4 exceeded 0.8.

### Protein–protein interaction network

We first utilized DrugBank to collect drug targets associated with AD treatment. Proteins targeted by drugs or ingredients approved for AD treatment were classified as drug targets. Drug information for these identified proteins was recorded. Next, we compiled information on potential targets that passed our FDR correction and CA validation. Subsequently, a protein–protein interaction (PPI) network was constructed using the STRING database (version 12) with a minimum interaction score threshold of 0.4. [28]. We aimed to investigate the interactions among the potential targets and to determine whether existing drug targets influence our predicted potential targets.

### Virtual screening

To identify small molecule compounds for potential targets, we used Schrödinger Maestro for structure-based virtual screening. For GSTP1 inhibitors, the virtual screening model was derived from the PDB database (PDB ID: 3DGQ). As to BIN1 inhibitors, the model of virtual screening is derived from the PDB database(PDB ID: 2FIC), and SiteMap was used to predict the most likely binding site for virtual screening since there is no small molecule binding site for BIN1. The Chemdiv 3D-Pharmacophore Based Diversity Library was prepared for virtual screening following energy optimization using Schrödinger's LigPrep module. These optimized compounds were then subjected to the Virtual Screening Workflow for molecular docking. Initial screening was performed using the High-Throughput Virtual Screening (HTVS) protocol to rapidly filter out compounds with low binding affinity. Next, compounds that passed the HTVS stage were subjected to Standard Precision (SP) docking to refine rankings based on more accurate scoring functions and to further narrow down the list of potential hits. A subset of the top-ranked ligands from SP docking was then subjected to Extra Precision (XP) docking to obtain highly accurate binding poses and docking scores. Finally, the most promising and representative compounds were selected through empirical visual inspection. The inclusion criteria were as follows: (1) Compounds representing different chemotypes to ensure a broad distribution across chemical space; (2) Compounds with high docking scores and reasonable docking poses; (3) Compounds that comply with Lipinski's Rule of Five. The exclusion criteria were as follows: (1) Compounds with poor synthetic feasibility; (2) Compounds difficult to modify or optimize. The Docking Score could quantitatively evaluate the likelihood and affinity of small molecule compounds binding to target proteins. The lower the score, the more stable the combination. For the objects of virtual screening, from the results of primary MR Analysis and CA, we initially identified BIN1 and GSTP1 because they have a positive causal relationship with AD and are driven by the genetic variations shared by identified plasma proteins and AD. Most proteins do not have natural agonists, so the small molecules obtained by virtual screening are generally protein inhibitors. Therefore, we decided to search for potential inhibitors of BIN1 and GSTP1.

### The impact of lifestyle on predicting protein targets

Additionally, we conducted an MR analysis on lifestyle factors and Alzheimer's disease-related proteins to determine which proteins can be regulated through lifestyle interventions. The MR analysis method was consistent with that described for the primary MR analysis.

## Results

### MR of the whole proteome identified 8 AD-related proteins

**Fig. 1:**
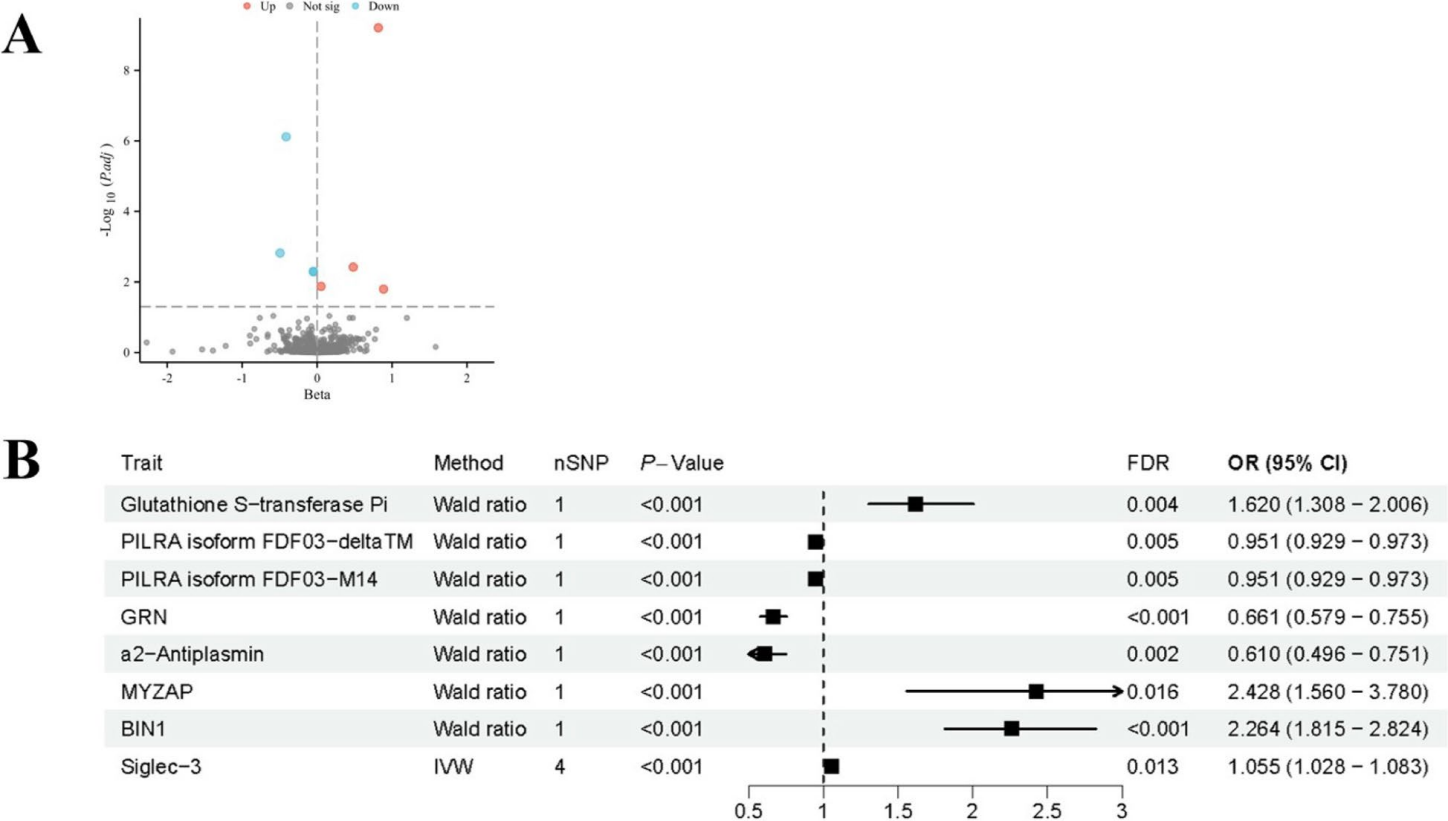
Analysis of the causal relationships between plasma circulating proteins and AD . **A**: The differences in plasma circulating protein levels. Up: Significantly increased proteins; Down: Significantly decreased proteins. **B**: The effect size of significant plasma circulating proteins on AD after FDR correction

In the cis-pQTL analysis of Alzheimer's disease, we preliminarily found a nominal association (P < 0.05) between 124 plasma proteins and Alzheimer's disease. However, after adjusting for false discovery rate (FDR), only 8 proteins showed a statistically significant correlation with Alzheimer's disease (FDR < 0.05, Fig. 1A-B), including Glutathione S-transferase Pi (GSTP1), PILRA isoform FDF03-deltaTM; PILRA isoform FDF03-M14; GRN; α2-Antiplasmin (SERPINF2); MYZAP; BIN1; Siglec-3. 4 of 8 proteins are risk factors of AD while the other 4 proteins are protective factors. To be specific, incremental GSTP1 (OR = 1.62; 95% CI, 1.31–2.01; P = 1.00 × 10^−5^), MYZAP (OR = 2.43; 95% CI, 1.56–3.78; P = 4.13 × 10^−13^), BIN1 (OR = 2.26; 95% CI, 1.82–2.82; P = 4.13 × 10^−13^) and Siglec-3 (OR = 1.05; 95% CI, 1.03–1.08; P = 6.19 × 10^−5^) increased the risk of AD, whereas elevated SERPINF2 (OR = 0.61; 95% CI, 0.50–0.75; P = 3.02 × 10^−6^), GRN (OR = 0.66; 95% CI, 0.58–0.76; P = 7.65 × 10^−7^), PILRA isoform FDF03-deltaTM (OR = 0.95; 95% CI, 0.93–0.97; P = 2.03 × 10^−5^), PILRA isoform FDF03-M14 (OR = 0.95; 95% CI, 0.93–0.97; P = 2.03 × 10^−5^) decreased the risk of AD. These results suggest that these 8 proteins may play important roles in the pathogenesis of Alzheimer's disease. Due to the presence of only 1–2 SNPs in most cases, there is no pleiotropy or heterogeneity. The detailed association results between all cis-pqtls and AD are attached (Supplemental Table 3).

### Proteins validated by colocalization analysis

**Fig. 2:**
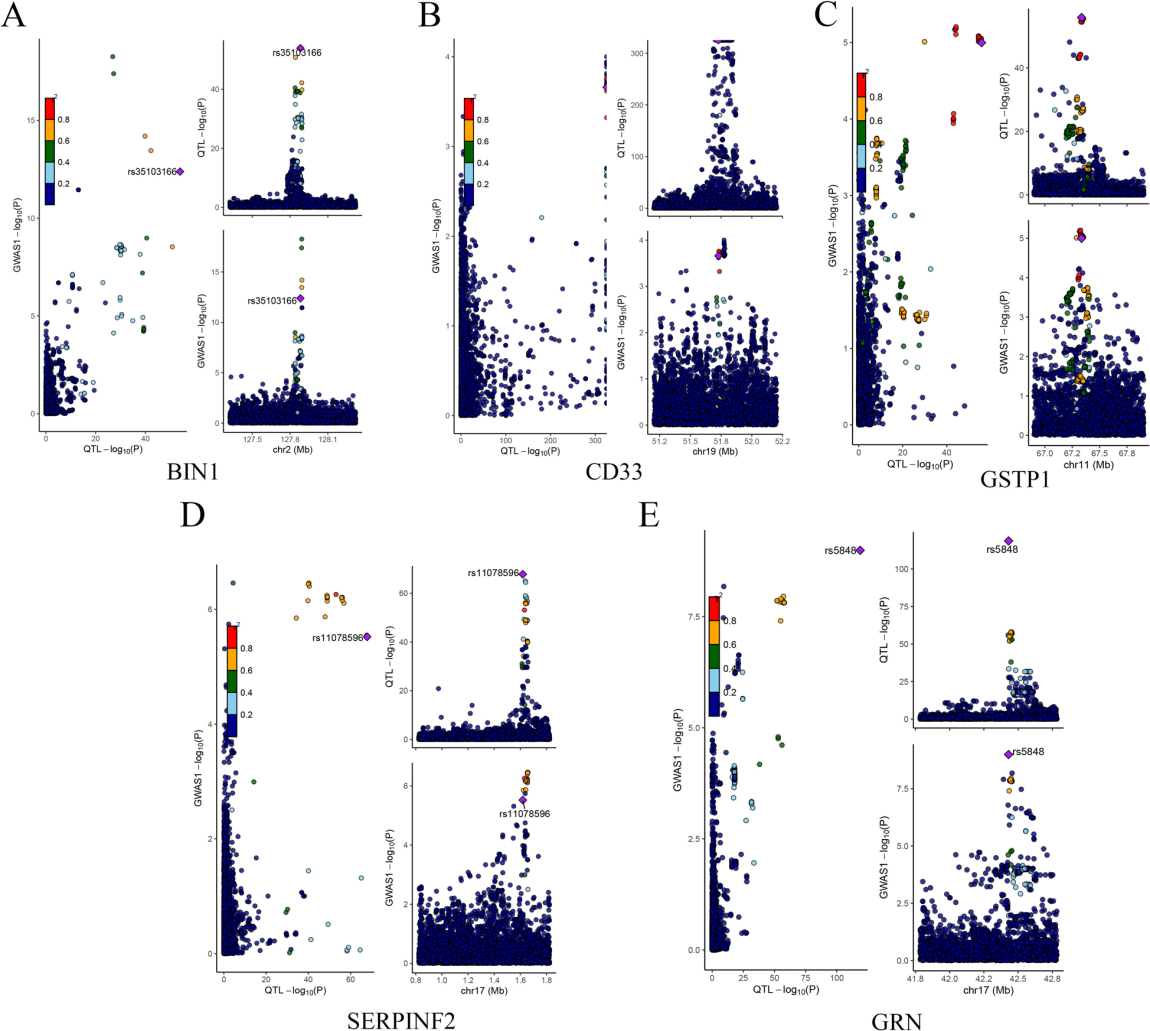
Analysis of co-localization **A**: BIN1; **B**: Siglec-3; **C**: GSTP1; **D**: SERPINF2; **E**: GRN

Among the 8 AD-related proteins, 5 proteins showed strong CA evidence at different windows, including BIN1, Siglec-3, GSTP1, SERPINF2, and GRN (PPH4 > 0.75/PPH3 + PPH4 > 0.8, Supplemental Table 4 and Fig. 2). To test whether the association between the five identified proteins and Alzheimer's disease is through reverse causality, we further performed the reverse MR. After FDR correction, no statistically significant association was found (Supplemental Table 5).

### Drug targets and PPI network construction

**Fig. 3:**
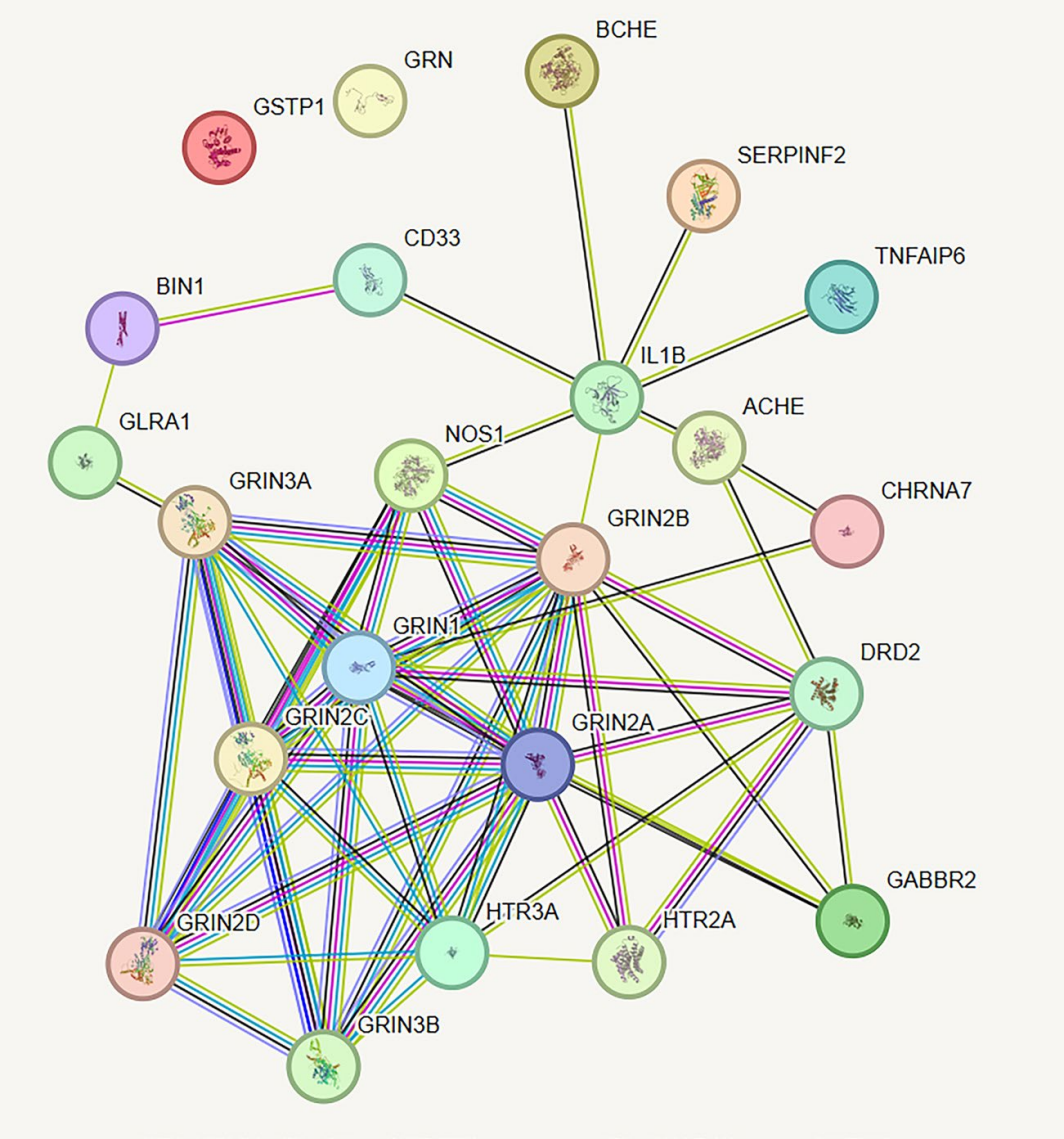
PPI network analysis of existing drug targets and predicted potential targets. Green lines neighborhood evidence; Blue lines: co-expression; Purple lines: protein homology; Yellow lines: text mining; Black lines: co-occurrence; Light blue lines: the interaction is curated from external databases

To further investigate the impact of existing drugs on AD, we identified five drugs currently used in AD treatment and their corresponding targets: Donepezil, Memantine Hydrochloride, Huperzine A, Galantamine, and Rivastigmine (Supplemental Table 6). Our analysis revealed that among the five selected targets, IL1B influences Siglec-3 and SERPINF2, while GLRA1 affects BIN1. Additionally, Siglec-3 and SERPINF2 interact with each other; however, GSTP1 and GRN are not influenced by any of these targets (Fig. 3). Consequently, we propose that Memantine Hydrochloride and Donepezil could serve as targeted therapies for the identified targets (Supplemental Table 7). Furthermore, the exploration of drugs targeting GSTP1 and BIN1 remains largely uncharted.

### Virtual screening

**Fig. 4:**
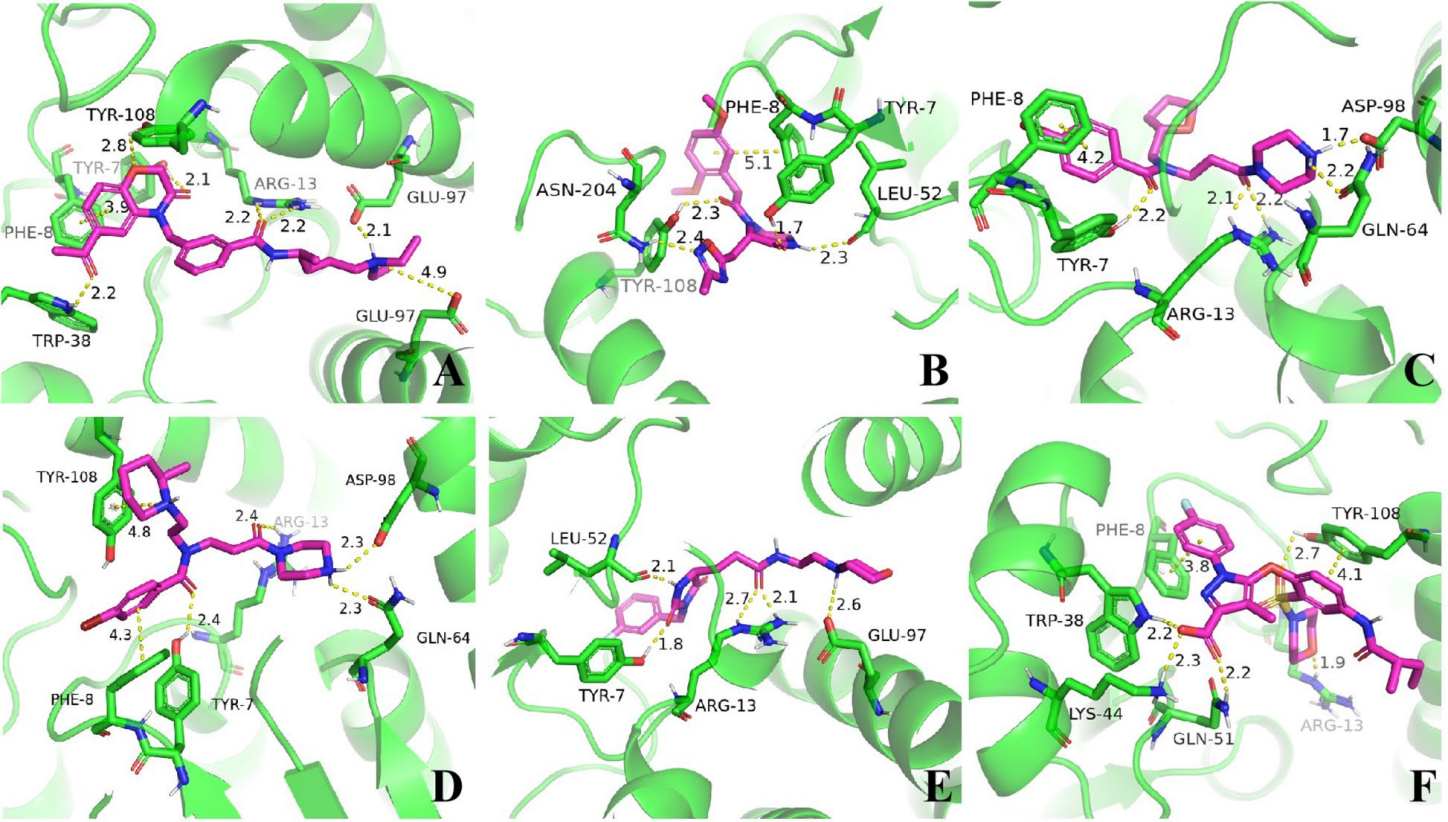
Binding patterns of promising small molecule compounds with GSTP1: **A**: V006-7774; **B**: SD12-0025; **C**: F594-0362; **D**: F594-0830; **E**: Y041-8058; **F**: V026-2799

For potential GSTP1 inhibitors, among the 720 compounds subjected to visual inspection, 93 exhibited docking scores below -7, while 625 had scores between -7 and -5 (Supplementary 8). Ultimately, six promising compounds were identified: V006-7774, SD12-0025, F594-0362, F594-0830, Y041-8058, and V026-2799 (Table 1). The 3D binding patterns of these compounds with GSTP1 are illustrated in Fig. 4. Compound V006-7774 forms hydrogen bonds with Tyr7, Tyr108, Trp38, and Arg13, pi-pi interactions with Phe8, and salt bridges with Glu97. Compound SD12-0025 forms hydrogen bonds with Tyr7, Leu52, Tyr108, Asn204, and Arg13, as well as a pi–pi interaction with Phe8. Compound F594-0362 forms hydrogen bonds with Arg13, Tyr7, and Gln51, pi-pi interactions with Phe8, and salt bridges with Asp98. Compound F594-0830 forms hydrogen bonds with Arg13, Tyr7, and Gln64, pi-pi interactions with Phe8, pi-cation interactions with Tyr108, and salt-bridging interactions with Asp98. Compound Y041-8058 forms hydrogen bonds with Tyr7, Leu52, and Arg13, as well as salt bridges with Glu97. Compound V026-2799 forms hydrogen bonds with Trp38, Arg13, Tyr108, and Gln51, pi-pi interactions with Phe8 and Tyr108, and salt bridges with Lys44.

**Table 1:** Identified Small Molecules as Potential GSTP1 and BIN1 Inhibitors

Targets	ID	Structure Formula	Molecular Weight (Dalton)	Docking Score
GSTP1	V006-7774		466.606	− 8.391
	SD12-0025		374.400	− 8.360
	F594-0362		425.354	− 7.865
	F594-0830		467.459	− 7.481
	Y041-8058		393.442	− 7.408
	V026-2799		559.598	− 7.151
BIN1	8012–8955		390.265	− 6.130
	Y500-7024		333.316	− 5.491
	Y042-2962		339.374	− 5.465
	7627–0037		282.302	− 5.325

**Fig. 5:**
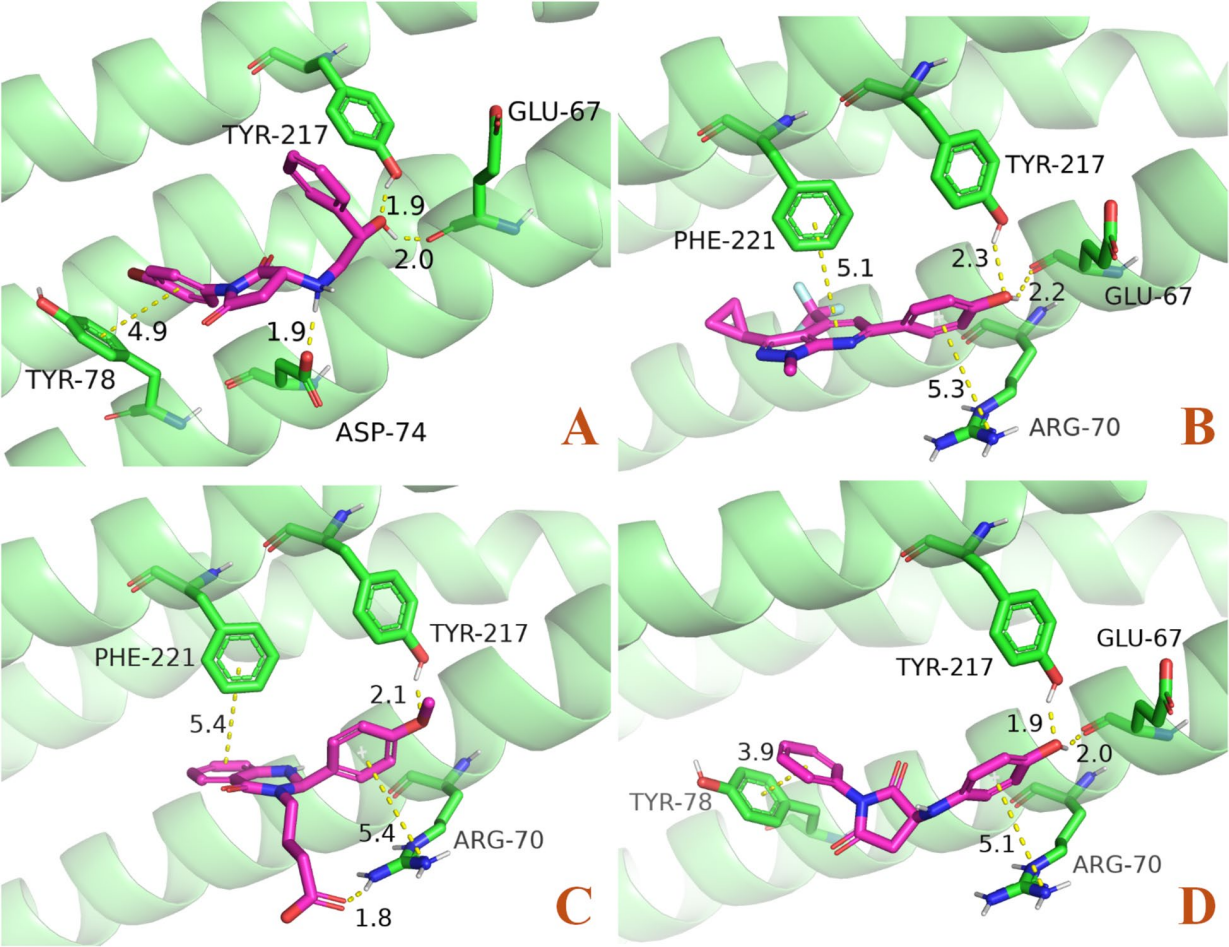
Binding patterns of promising small molecule compounds with BIN1: **A**: 8012-8955; **B**: Y500-7024; **C**: Y042-2962; **D**: 7627-0037

As to potential BIN1 inhibitors, 268 compounds underwent visual inspection, and 40 had a docking score below -5. (Supplementary 9). Finally, four promising compounds were identified: 8012–8955, Y500-7024, Y042-2962, and 7627–0037 (Table 1). The 3D binding patterns of these compounds with BIN1 are illustrated in Fig. 5. The compound 8012–8955 can form hydrogen bonds with Tyr217 and Glu67, pi-pi interactions with Tyr78, and salt Bridges with Asp74. Y500-7024 can form hydrogen bonds with Tyr217 and Glu67, pi-pi interactions with Phe221, and pi-cation interactions with Arg70. Y042-2962 can form hydrogen bonds with Tyr217, pi–pi interaction with Phe221, salt bridges, and pi-cation interactions with Arg70. 7627–0037 can form hydrogen bonds with Tyr217 and Glu67, pi-pi interactions with Tyr78, and pi-cation interactions with Arg70.

### Impact of lifestyle factors on Ad-related proteins

Among the 17 lifestyle factors and 5 proteins associated with AD identified previously through MR, 3 healthy lifestyle factors (Crisp intake, Dried fruit intake, and Smoking status: Never) were associated with 2 proteins (Supplemental Table 10, Supplemental Fig. 1). Specifically, crisp intake (Beta = − 0.30, P = 0.02) and smoking status (Never) (Beta = -0.22, P = 0.04) are negatively related with BIN1 while dried fruit intake(Beta = -0.23, P = 0.016) is negatively associated with SERPINF2.

## Discussion

The detection of plasma proteins may indicate broader systemic processes associated with AD pathophysiology, including neuroinflammation, oxidative stress, and metabolic dysregulation. As such, plasma proteins can serve as valuable biomarkers of disease states, offering accessible targets for therapeutic intervention. Although the primary pathological manifestations of AD occur within the brain, systemic alterations—such as those measurable in plasma—can influence disease progression. Consequently, plasma proteins present a potentially modifiable therapeutic pathway, particularly for strategies targeting the peripheral regulation of neuro degenerative processes.

In this study, we employed MR to identify plasma circulating proteins associated with AD, with five (GSTP1, BIN1, Siglec-3, SERPINF2, GRN) showing statistically significant associations with AD and strong evidence of CA, suggesting their potential causal roles in AD pathogenesis. Protein–protein interaction (PPI) analysis revealed interactions between identified targets and current AD drug targets, highlighting GSTP1 and BIN1 as promising new targets for drug development. We preliminarily conducted virtual screening and identified several potential small-molecule inhibitors.

Previous research results have shown that smoking is associated with AD neuropathology in preclinical models and humans. Smoking related oxidative stress in the brain is a potential mechanism that promotes AD pathology and increases AD risk [29]. And our results found that quitting smoking may prevent AD by affecting BIN1 levels, which is consistent with previous findings. Previous studies have yielded contradictory results regarding the impact of dried fruit intake on AD. Deng's study [30] found that an increase in dried fruit intake was associated with a decrease in AD risk, while Liao et al.'s study found a positive correlation between an increase in dried fruit intake and AD risk [29]. And our reduction in dried fruit intake may lower the risk of AD by affecting SERPINF2. We believe that the reason for this difference may be due to differences in sample size, or the types of dried fruits studied.

A “causality” identified by MR might be horizontal pleiotropy, genetic confounding due to linkage disequilibrium (LD), or reverse causality. To limit the bias from horizontal pleiotropy, we only used cis-pQTLs as the instruments, given their direct role in the transcription and/or translation of related genes [31]. In addition, Bayesian CA was also used to exclude the bias introduced by LD. With PPH4 exceeding 0.75 in different windows or when PPH3 + PPH4 exceeding 0.8 as the critical threshold for posterior probability, the five proteins identified were likely to share the same variant of AD [32]. Bidirectional MR was conducted in the study and no proteins showed reverse causality.

Growing evidence suggests that variations and expression levels of multiple genes play a critical role in neuroinflammation and neuro degenerative processes within the pathophysiology of AD. GSTP1 is the most widely studied member of the GST family [33]. It is supposed to involve in metabolism, detoxification and elimination of potentially genotoxic foreign complexes, metabolizes a variety of carcinogenic compounds and protects cells against DNA damage and canceration [34]. However, it may play the villain in AD. In 2016, GSTP1 Ile105Val polymorphism was found to be associated with increased risk of AD [35]. Then, in 2017, a study reported that GSTP1 was involved in anxiety and depression behaviors in 10-month-old triple transgenic mice of AD, and melatonin could serve as a potential candidate drug to improve the neuropsychiatric behaviors in AD via modulating the expression of the GSTP1 [36]. Our study further proved that GSTP1 is positively associated with AD in humans, with one standard deviation (SD) increase raising the risk by 62%. BIN1, its down-regulation was founded to be related to cancer progression and also correlates with ventricular cardiomyopathy and arrhythmia preceding heart failure while increased BIN1 expression maybe linked with increased susceptibility for AD [37]. A study investigated the role of BIN1 in regulating neuroinflammatory responses in microglia by employing various molecular techniques, including immunostaining, RT-PCR, siRNA knockdown, and Cre-lox conditional deletion in both mouse and human models, with findings analyzed through gene expression panels, flow cytometry, and pathway analysis. The result shows that BIN1 is primarily involved in clathrin-mediated endocytosis and has been implicated in the regulation of neuroinflammation through its role in microglial activation [38]. Our study quantitatively underscores the enormous risk of BIN1 protein level, showing that each SD increase raises the likelihood of AD by 126%. Furthermore, Siglec-3 -related research has become a new hot topic. Siglec-3 is an immunomodulatory receptor expressed on microglia and is implicated in the inhibition of microglial-mediated clearance of Aβ. [39] Our study demonstrated the positive association between increased Siglec-3 protein level and AD risk, this could be explained as a stronger inhibitory ability on Aβ clearance mediated by microglia, however, the effect size is relatively small. For every one SD increase of Siglec-3 in plasma concentration, the risk of AD merely increases by 3%. In contrast to these risk factors, GRN is a multifunctional protein involved in the regulation of neuroinflammation and the promotion of neuronal survival. Reduced GRN levels have been linked to increased neuroinflammation and neuronal death, contributing to the pathogenesis of AD [40]. GRN was identified as a protective factor as well in our research, with each standard deviation increase in plasma concentration reducing AD risk by 34%. In addition, a study assessed the methylation state of the brain's DNA in relation to AD using 708 prospectively collected autopsied brains [41]. By comparison, although SERPINF2 has been mentioned in previous literature, its specific causal relationship with AD has not been clearly validated. Therefore, our study provides causal evidence for SERPINF2 protein as a potential regulatory factor for Alzheimer's disease for the first time.

Despite advancements in therapies in recent years, current treatment options for AD remain challenging. The pathophysiology of AD involves multiple complex processes, with glutamatergic excitotoxicity recognized as a significant contributor to neuronal damage and death. The NMDA receptor, a subtype of glutamate receptors, is central to synaptic plasticity and cognitive functions, including learning and memory. In pathological conditions, particularly in AD, excessive glutamate release leads to sustained activation of NMDA receptors. This chronic activation results in an influx of calcium ions into neurons, triggering a cascade of intracellular events that culminate in neuronal injury and apoptosis—a phenomenon known as excitotoxicity [42]. Memantine Hydrochloride exerts its therapeutic effects by acting as a non-competitive antagonist at the NMDA receptor, specifically binding to the NMDA receptor subunit 2B (encoded by the GRIN2B gene). This action selectively blocks the prolonged calcium ion influx associated with excitotoxicity while preserving normal physiological synaptic transmission, which is essential for cognitive function [43]. Protein–protein interaction analysis based on the STRING database identified GLRA1 as another target of Memantine Hydrochloride. Moreover, GLRA1 and BIN1 appear to be connected within the network, suggesting a potential interaction or co-expression relationship. BIN1 is involved in membrane dynamics, particularly in endocytosis and membrane curvature, whereas GLRA1 is a component of the glycine receptor, a ligand-gated ion channel involved in inhibitory neurotransmission [38]. The association between GLRA1 and BIN1 may indicate a functional relationship in synaptic activity or neuronal signaling. BIN1's role in membrane remodeling could influence the localization or trafficking of GLRA1, thereby affecting glycinergic neurotransmission. Additionally, Donepezil, a medication primarily used to treat cognitive symptoms in AD, mainly targets and inhibits acetylcholinesterase, an enzyme responsible for the breakdown of acetylcholine in the synaptic cleft [44]. Acetylcholine plays a critical role in memory formation and cognitive function, leading to improvements in symptoms such as memory, attention, and other cognitive abilities. Interestingly, in our study, Donepezil was also determined to act on the IL1B target, and the correlations between IL1B and Siglec-3, SERPINF2 were then identified. IL1B is a critical pro-inflammatory cytokine that plays a central role in the inflammatory response and is often involved in the pathogenesis of various diseases, including autoimmune disorders [45]. The interaction between IL1B and Siglec-3, a sialic acid-binding immunoglobulin-like lectin primarily expressed on myeloid cells, suggests an underlying regulatory mechanism in which Siglec-3 modulates IL1B-driven immune responses. This interaction potentially influences the activation state of myeloid cells during inflammation, highlighting the importance of Siglec-3 in immune regulation [46]. SERPINF2, also known as alpha-2 antiplasmin, is a serine protease inhibitor that primarily inhibits plasmin, the major enzyme responsible for fibrinolysis [47]. The interaction between IL1B and SERPINF2 implies a role in modulating inflammation-driven proteolytic activity, which could be particularly relevant in pathological conditions where inflammation and fibrinolysis are closely linked, such as in tissue repair, or certain inflammatory diseases.

Furthermore, to the best of our knowledge, this is the first study to discover the positive causal association between GSTP1 and AD and no interactions between selected AD drug targets and GSTP1 were observed. GSTP1 plays a critical role in cellular detoxification by catalyzing the conjugation of glutathione to various electrophilic compounds. Structurally, GSTP1 is a homodimer, with each subunit comprising approximately 210 amino acids. The enzyme's structure features a highly conserved N-terminal domain that binds glutathione and a more variable C-terminal domain responsible for interacting with substrates [48]. The active site of GSTP1 is formed by a combination of residues from both domains, creating a catalytic pocket essential for its enzymatic activity. In fact, this detoxifying enzyme is over-expressed in erythrocytes when unusual amounts of toxins are present in the body [49]. Therefore, the reverse causality between GSTP1 and AD cannot be completely ruled out although no significant results were indicated in the reverse MR. More research is needed to elucidate the specific mechanism between GSTP1 and AD. Nonetheless, we still did some virtual screening work to search for GSTP1 inhibitors as it is particularly promising in the context of cancer therapy, not just for AD treatment. The overexpression of GSTP1 has been associated with resistance to chemotherapy, as it can detoxify chemotherapeutic agents, reducing their efficacy [50]. Therefore, inhibitors targeting GSTP1 could potentially enhance the effectiveness of chemotherapy by preventing the enzyme from neutralizing these drugs. Six potential lead compounds for GSTP1 inhibitors were identified in our study, and more experiments including Enzyme-Linked Immunosorbent Assay (ELISA) and Surface Plasmon Resonance (SPR) are needed to further evaluate these intermolecular interactions.

Currently approved AD treatment drugs only provide symptomatic benefits. Emerging epidemiological and clinical studies suggest that lifestyle changes offer an alternative therapeutic route for slowing cognitive decline and AD development [51]. For example, adherence to a Mediterranean diet, including core foods such as fish, olive oil, fruit, and green leafy vegetables, may reduce the risk of AD [52]. Crisp foods generally refer to vegetables and fruits, which have a crisp and refreshing taste and are rich in fibre, vitamins, and minerals. In our study, more crisp food intake was significantly associated with lower BIN1 levels and thus could be a protective lifestyle factor of AD. This may be related to the involvement of vitamins in crisp foods in alleviating oxidative stress processes. Conversely, dried fruit intake was found to be negatively associated with SERPINF2, thus a risk lifestyle factor of AD. This result is consistent with a recently published MR study [53], and our findings may contribute to explaining the mechanism of more dried fruit intake increasing AD risk. For addictive behaviours, drinking and smoking are the focus of attention. Literature indicates that former/active smoking is related to a significantly increased risk for AD [29], while the relationships between alcohol drinking has dose-related associations with AD [22]. In our study, alcohol intake was not found to be related to AD, while never smoking is identified as a protective lifestyle factor for AD, with never-smokers having relatively lower BIN1 protein levels.

This study possesses several strengths. First, it employs a comprehensive methodology that combines MR, CA, PPI network construction, and virtual screening, allowing for a thorough investigation of potential therapeutic targets for AD. Second, five plasma proteins were identified as targets significantly associated with AD, particularly GSTP1 and BIN1, which have not been extensively studied in the context of AD. Third, the successful virtual screening identified six promising compounds as potential GSTP1 inhibitors, highlighting the utility of computer-aided drug discovery in identifying therapeutic candidates. Furthermore, the study offers valuable insights into the influence of lifestyle factors on AD risk, suggesting that behaviors such as increased intake of crisp foods and smoking cessation may mitigate AD risk through their impact on specific proteins. Finally, the study’s rigorous validation techniques, including Bayesian CA and reverse MR, enhance the credibility of these findings, demonstrating the interdisciplinary impact of integrating genetic, proteomic, and virtual screening approaches to advance the understanding of AD pathogenesis and identify new therapeutic strategies.

However, this study is not without limitations. First, all investigated plasma circulating proteins had only one or two cis-acting SNPs and lacked trans-pQTLs, which limited the application of various analyses, including alternative MR algorithms, as well as tests for heterogeneity and pleiotropy. Second, this study primarily utilized genetic data from European populations, and it was difficult to generalize the results to other ancestries. Variations in genetic architecture across populations could result in different associations between cis-pQTLs and AD, thereby affecting the broader applicability of the study's results. Third, despite the identification of highly promising small-molecule compounds through virtual screening, the lack of experimental validation due to inherent limitations in conditions remains a drawback. Finally, the datasets selected in this article are all European datasets, and the final research results may not be applicable to non-European populations. In future research, we will continuously expand the data sources to improve the generalizability and applicability of the research results.

## Conclusion

In summary, this study identified five plasma proteins (GSTP1, BIN1, Siglec-3, SERPINF2, and GRN) as potential therapeutic targets for AD using MR and CA. GSTP1 and BIN1 were highlighted as novel promising targets, with virtual screening identifying a total of ten potential inhibitors. Additionally, lifestyle factors, including crisp food intake, dried fruit intake and smoking cessation were found to influence AD risk through their effects on specific proteins. These findings provide a strong foundation for further research into targeted therapies and preventive strategies for AD, though more experimental validation is needed.

## Supplementary Information

Additional file 1.

Additional file 2.

Additional file 3.

Additional file 4.

Additional file 5.

Additional file 6.

Additional file 7.

Additional file 8.

Additional file 9.

Additional file 10.

## Data Availability

The original contributions presented in the study are included in the article/Supplemental Table, and further inquiries can be directed to the corresponding author.
