# Supplementary figures and images for: Uncovering leading compounds for alzheimer’s disease treatment: mendelian randomization and virtual screening insights into plasma protein modulation
Source: Biol Res. 2025 Apr 5;58:19. doi: 10.1186/s40659-025-00598-2 (PMC11971886; doi:10.1186/s40659-025-00598-2)

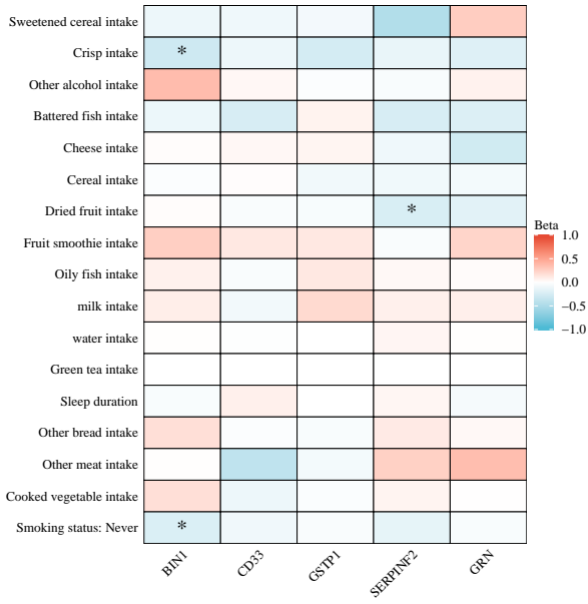

Supplement: Supplementary file 2 — Additional file 2. [file 40659_2025_598_MOESM2_ESM.pdf]
